# Medicine shortages: impact behind numbers

**DOI:** 10.1186/s40545-023-00548-x

**Published:** 2023-03-14

**Authors:** Doerine J. Postma, Kim Notenboom, Peter A. G. M. De Smet, Hubert G. M. Leufkens, Aukje K. Mantel-Teeuwisse

**Affiliations:** 1 Division of Pharmacoepidemiology and Clinical Pharmacology, Utrecht Institute for Pharmaceutical Sciences (UIPS), Utrecht University, Universiteitsweg 99, 3584 CG Utrecht, The Netherlands; 2 Royal Dutch Pharmacists Association, The Hague, The Netherlands; 3 Dutch Medicines Evaluation Board, Utrecht, The Netherlands; 4 Departments of IQ Health Care and of Clinical Pharmacy, Radboud University Medical Centre, Radboud Institute for Health Sciences, Nijmegen, The Netherlands

**Keywords:** Medicine shortages, Impact, Framework, ECHO model, Patient outcomes

## Abstract

**Introduction:**

Current research to assess the impact that medicine shortages have on patients is limited to general aspects, such as the prevalence of shortages and product characteristics. The aim of this study is to assess the overall impact that medicine shortages have on economic, clinical, and humanistic outcomes.

**Methods:**

A cohort of all known products in shortage in the Netherlands between 2012 and 2015 were characterized by their route of administration, anatomical therapeutic chemical class, and whether they were originator or generic products. A representative sample of 324 shortages (18% of all shortages) was rated as having low, medium, or high impact on the five elements that determine the impact of shortages on patients: availability of an alternative product, underlying disease, susceptibility of the patient, costs (for patients and society at large), and number of patients affected. Ratings were converted into numerical scores per element and multiplied to obtain an overall impact score.

**Results:**

Two elements were most frequently rated as having a high impact: disease (29%) and costs (20%). Nearly half of the shortages (47%) rated high on at least one element, while nearly 10% rated high on multiple elements. Thirty percent of the shortages rated high on direct impact, which is represented by these elements: alternative product and disease. An additional 17% of the shortages rated high on indirect impact, which is represented by these elements: costs, susceptibility, and number of patients. High impact scores could not significantly be attributed to characteristics of the products in shortage.

**Conclusions:**

An assessment of the medicine shortages’ impact using a framework based on economic, clinical, and economic outcomes showed that all three outcomes affect the overall impact that medicine shortages have on patients.

**Supplementary Information:**

The online version contains supplementary material available at 10.1186/s40545-023-00548-x.

## Introduction

Medicines are an essential part of medical care. They improve patients’ health and quality of life [1]. However, global concerns have developed surrounding ensuring their continuous supply [2, 3]. Supply chain management has always been an area of concern for patients and health care professionals in low- and middle-income countries [4]. Since the beginning of this era, reports on medicine unavailability in high-income countries have started to increase [5, 6], ending a decades-long period of seemingly continuous access to medicines in high-income countries. In fact, the temporary medicine unavailability has become “the new normal” [7] or “business as usual” [8]. With the COVID-19 pandemic, this phenomenon became more commonplace as shortages of essential medicines increased [9–11], bringing the long-standing vulnerability of the medical product supply chain into sharp focus [12].

Not all unavailability of medicines can be classified as a medicine shortage. Several definitions of medicine shortages exist, depending on the stakeholder and the purpose of the definition [13]. For example, health care professionals and patients consider a shortage “any inability to supply a specific medicinal product to an individual patient within a defined period” [14]. For the purpose of notification and detection of shortages by marketing authorization holders (MAHs), authorities define unavailability as a shortage when “supply does not meet demand at a national level”, affecting the whole patient population [15]. Different definitions used by stakeholders result in different numbers of medicine shortages. A recent study also showed that national authorities in European countries collect information on and report shortages differently. Therefore, quantitative data cannot be used to make a direct comparison between countries [16]. Also, a focus on numbers of medicine shortages suggests that all shortages have a uniform impact. However, some shortages have a larger impact than others.

Medicine shortages impact many stakeholders in the pharmaceutical value chain. Health care professionals, manufacturers and authorities need to redirect their time to solve medicine shortages. Patients are affected directly: they are the ones who need to switch to another label with the same active substance (generic substitution), switch to medicines containing another active substance (therapeutic substitution), postpone treatment, or have treatment denied. The impact on other stakeholders also affects patients, but in a more indirect way. Health care professionals have less time for medical care [17], manufacturers are losing part of their profit and may look for other means to recoup return on investment [18], and medicine regulatory agencies and other authorities must increase their capacity for preventing and mitigating the effects of medicine shortages [19]. Whereas the direct impact on patients is clear, the indirect impact is often overlooked.

With increasing numbers of shortages, identifying shortages having high-patient impact may be helpful in mitigating the impact of shortages on patients in a timely manner. Efforts should target shortages having the highest patient impact. One method to assess the impact on patients is the economic, clinical, and humanistic outcomes (ECHO) model. The ECHO model balances outcomes to ensure that one outcome type is not maximized at the expense of another [20, 21]. Product characteristics, such as therapeutic class, route of administration, and whether they are patented or generic [19, 22], are often related to clinical effects, such as the presence of an adequate alternative product. It is generally assumed that shortages of antibiotics, chemotherapies, and products for parenteral use have a high impact on patients since these medicines are difficult to substitute. Besides the clinical effects, medicine shortages also have economic effects [22], such as increased prices and personnel costs [22–25]. While a few publications reviewed the costs of specific shortages, they were incomplete since personnel costs were excluded [26–28]. Studies on humanistic effects, such as patient related factors that determine the resolution of medicines shortages [29] or patients’ concerns as a result of the shortage, are even more scarce especially on specific medicine shortages [30]. Indirect impact and health outcomes are seldom reported in the literature [5].

The economic, clinical, and humanistic aspects of medicine shortages have been elaborated in general [30, 31], but not assessed for actual shortages. An ECHO-based framework was developed to assess and visualize the impact of medicine shortages on patients. This framework provides an opportunity to combine all outcomes. This framework was founded on several learning cases [32], but has not yet been applied to a large set of shortages.

The aim of this study is to assess the impact of medicine shortages on patients using the previously developed and piloted objective ECHO-based framework. Assessing the impact of shortages on patients may help signal and identify shortage trends and prioritize efforts to mitigate their impact.

## Methods

### Study design

For a cohort of shortages, the products in shortage were categorized by their route of administration, their anatomical therapeutic chemical (ATC) classification, and whether they were the patented originator or a generic (unpatented) product. The shortages were rated on five elements that determine the impact of shortages on patients: alternative product, disease, susceptibility, cost, and number of patients [32]. These elements were traced back to economic, clinical and humanistic outcomes.

### Study population

Data on a cohort of all known shortages in the Netherlands starting between 2012 and 2015 were collected. This data set was complete and recorded in detail. Moreover, the cases were closed, so insights into all aspects of a shortage were possible to ascertain. This dataset has been described in detail in previous research [33]. In short, the dataset includes all shortages voluntarily reported by Dutch pharmacists to the Dutch pharmacy practice (Koninklijke Nederlandse Maatschappij ter bevordering der Pharmacie, KNMP). The information on these shortages was publicly available at that time and consisted of information on the shortage itself and a possible solution for patients. The dataset was complemented with mandatory shortage notifications from MAHs to the Dutch authorities (Dutch Medicines Evaluation Board; MEB and Dutch Health and Youth Care Inspectorate; Inspectorate).

### Definitions and characteristics

A shortage was defined as “a marketing authorization (MA) for human use that is nationally unavailable for at least two weeks”. This included permanent and temporary changes to the marketing status. A period of unavailability of less than 2 weeks is likely to be mitigated by stock that is still present in the supply chain (with the patient, in another pharmacy, or at a wholesaler) and therefore has been chosen as the cut-off point by the KNMP. Parallel import products were excluded because fluctuations in their availability are inherent. Homeopathic medicines, herbal medicines, and unregistered products, such as raw materials, pharmacy preparations, and food additives, were also excluded. All shortages were either reported or validated by the MAH. If a shortage was reported within 30 days of the resolution of a previous shortage, it was considered the same shortage. After 30 days, it was considered a new shortage.

Shortages were reported per calendar year based on when the shortage started. The products in shortage were categorized by their route of administration, according to the Standard Terms of the European Directorate for the Quality of Medicines & HealthCare [34], by their WHO anatomical therapeutic chemical (ATC) classification [35], and whether they were the patented originator or a generic (unpatented) product.

### Impact—scoring elements

The shortages were rated on five elements that determine the impact of shortages on patients, according to the previously published framework [32] (see Box 1 for a summary). These five elements are alternative product, disease, susceptibility, cost, and number of patients. Each individual element was rated according to the framework as follows: low-impact score (1 point), moderate-impact score (2 points), or high-impact score (3 points). For examples of elements and their ratings, see Additional file 1. If one element consisted of several aspects (e.g., susceptibility) that were rated differently, the highest rating was taken as the overall score for that element. The rating of the elements was assessed by one researcher. Inconclusive elements were discussed with a second researcher.

For the element “alternative product”, the proposed solution published by KNMP at the time the shortage started was analyzed. For this solution, a standardized framework was used, as described in Box 2. If the shortage was not published on this website, since it was only reported to the authorities, KNMP’s system was applied retrospectively.

For the element “disease”, the indication from the patient leaflet and the corresponding disability weights according to WHO [36] were analyzed. If WHO classified a disease as “mild”, “moderate”, and “severe” with different scores—depending on the progression of the disease—the disease was regarded as “moderate”.

The element “susceptibility” comprised two aspects: vulnerability of the patient population and trust in the alternative therapy. For vulnerability, the age range was rated based on the patient leaflet. For trust in the alternative therapy, KNMP Farmanco’s data were analyzed for notes on patient contact, media attention, or reports on patient fora.

The costs were determined from the moment the shortage started. For the costs of a shortage, list prices of medicines were collected from the Dutch national medicine database (G-Standaard) [37]. The costs of the alternative treatment were compared to the treatment in shortage (percentage). The median and the interquartile range (IQR) were also calculated. Medicine costs above the IQR3 were regarded as “high” and below the IQR1 as “low”. Personnel costs were based on the amount of time spent on a shortage. For instance, pharmacists spend time communicating with other health care professionals and patients [38]. With an increasing impact level of the element “alternative product”, the personnel costs were likely to increase. For example, it takes some time to explain the change in a label to a patient. Additionally, a proposal for a therapeutic substitution, with potentially different (side) effects, takes even more time to explain since the pharmacist must talk to the treating physician and provide a more extensive explanation to the patient. The absence of another therapy probably consumes the most time, necessitating extensive deliberation between the pharmacist and the treating physician and between the pharmacist and the patient. Therefore, the element “personnel costs” were rated at the same level as the element “alternative product”.

Finally, the “number of patients affected” was estimated based on the number of people who used the product during the year prior to the shortage. For products that were dispensed in public pharmacies, these data were obtained from the Dutch Foundation for Pharmaceutical Statistics. The number of patients for hospital products was rated as “moderate” by default. If the patient leaflet stated an orphan indication, the number of patients was rated “low”.

### Impact—scoring outcomes according to the ECHO model

**Fig. 1:**
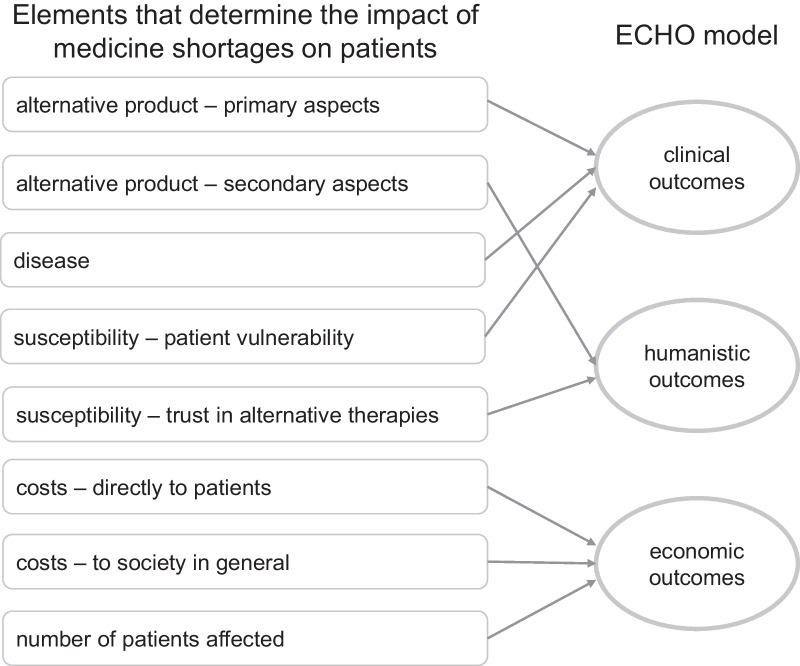
Elements that determine patient impact of shortages traced using the ECHO model

The five identified elements can be traced using the ECHO model [21], in which each outcome is represented by two or three elements (Fig. 1):

- Economic outcomes: costs and number of patients affected.
- Clinical outcomes: disease, alternative product (primary aspects), and susceptibility (vulnerability).
- Humanistic outcomes: alternative product (secondary aspects) and susceptibility (trust in alternative therapy).

If the underlying elements of one of these three outcomes were rated differently, the highest rating was taken as the overall rate for that outcome.

### Sample selection

A sample of 319 or more cases is representative of the case population of 1844, with a confidence level of 95%, a margin of random error of ± 5%, and a population proportion of 50%. The sample of shortages was randomly selected from the entire cohort using an online sampler.

### Statistics

To compare the characteristics of the total population of products in shortage and the randomly selected sample, a Chi-square test was performed. *P*-values < 0.05 were considered significant.

To calculate the overall impact of a medicine shortage, each of the five elements was rated and thus scored. The assigned scores were multiplied. Theoretically, the highest overall score on all elements would be 243 (3^5^). The median of the overall scores and IQR were calculated per ATC class, route of administration, and if the product was an originator or a generic product. To compare these overall scores, the Kruskal–Wallis and Dunn tests were performed. *P*-values < 0.05 were considered significant. Statistical analyses were performed using IBM SPSS 28, and all other analyses were performed using Microsoft Excel, version 16.35.

Box 1: Five elements as core drivers behind the impact of medicine shortages on patients In a previous study [32], five elements were identified as core drivers behind the impact of medicine shortages on patients:

1. Alternative product—impact on patients depends on the following:
  Primary aspects, such as active substances, licensing, and on- and off-label use.
  Secondary aspects, such as different route of administration, strength, or concentration.
2. Disease—to quantify health levels associated with a non-fatal outcome by means of disability weights.
3. Susceptibility—impact of alterations in medication depends on the following:
  Patient vulnerability.
  Trust in alternative therapies.
4. Costs are attributed to the following:
  Directly to patients, such as (partial) reimbursement.
  To society in general due to, for instance, higher prices for alternative medicines and personnel costs for additional work.
5. Number of patients affected: affecting the public concern.

Examples of the elements behind the impact of the shortage on patients are presented in Additional file 1.

Box 2: Data sources explained In 2004, the KNMP started publishing information about ongoing medicine shortages on a public website (www.farmanco.knmp.nl). The KNMP receives voluntary reports from pharmacists, which are verified by the MAHs before publication. The published information consists of data on the shortage itself, such as the date the shortage is expected to be resolved, and possible solutions for patients. Preferably, a shortage is solved by an authorized product that contains the same active substance and route of administration, a so-called generic substitution. If this is not (sufficiently) available, an authorized product with another substance for the same indication, a so-called therapeutic substitution, is advised. If this is not (sufficiently) available, unlicensed products (in the Netherlands) are listed. Examples of unlicensed products are pharmacy preparations and imported products. Inevitably, a suitable therapy may be absent.

National competent authorities receive mandatory reports on shortages from MAHs as per the Medicines Act of 2007. Until 2017, MAHs have had to report foreseen shortages to the Dutch MEB and unforeseen shortages to the Dutch Inspectorate. In 2017, the Dutch authorities launched one central point for the submission of notifications, the Medicine Shortages and Defects Notification Center (www.medicineshortagesdefects.nl).

## Results

### Characteristics of products in shortage

In the Netherlands, 1844 shortages were reported to pharmacy practice and authorities between 2012 and 2015. The number of shortages increased from 387 in 2012 to 548 in 2015. Over half of the shortages (56.7%) were related to oral products, and three out of ten (30.0%) were related to parenteral products. Less than 5% involved other routes of administration. Shortages most often occurred in medicinal products for the nervous system (ATC class N; 18.1%), anti-infectives for systemic use (ATC class J; 16.9%), and medicinal products for the cardiovascular system (ATC class C; 13.0%). The products in shortage involved originator products (54.8%) and generic products (45.2%).

The impact was rated for 324 randomly selected shortages (18% of all shortages). No significant difference was observed between the overall data set and the sample (all *p*-values > 0.05). An overview is presented in Table 1.

**Table 1:** Characteristics of shortages in the Netherlands 2012–2015, *n* (%)

Characteristics		All shortages *n* = 1844 (%)	Sample of shortages *n* = 324 (%)	*p*-value of Chi-square test
*Year shortage started*				0.207
2012		387 (21.0)	58 (18.2)	
2013		439 (23.8)	77 (24.1)	
2014		470 (25.5)	77 (24.1)	
2015		548 (29.7)	112 (35.1)	
*Route of administration*				0.322
Oral		1046 (56.7)	168 (52.7)	
Parenteral		554 (30.0)	104 (32.6)	
Nasal/inhalation		42 (2.3)	8 (2.5)	
Cutaneous		77 (4.2)	13 (4.1)	
Rectal		25 (1.4)	8 (2.5)	
Ocular		65 (3.5)	16 (5.0)	
Other		35 (1.9)	7 (2.2)	
*ATC class*				0.325
A	Alimentary tract and metabolism	168 (9.1)	27 (8.5)	
B	Blood and blood forming organs	91 (4.9)	17 (5.3)	
C	Cardiovascular system	240 (13.0)	38 (11.9)	
D	Dermatologicals	83 (4.5)	15 (4.7)	
G	Genito-urinary system and sex hormones	77 (4.2)	13 (4.1)	
H	Systemic hormonal preparations, excl. sex hormones and insulins	84 (4.6)	11 (3.4)	
J	Antiinfectives for systemic use	311 (16.9)	59 (18.5)	
L	Antineoplastic and immunomodulating agents	148 (8.0)	26 (8.2)	
M	Musculo-skeletal system	85 (4.6)	15 (4.7)	
N	Nervous system	333 (18.1)	56 (17.6)	
P	Antiparasitic products, insecticides and repellents	4 (0.2)	3 (0.9)	
R	Respiratory system	88 (4.8)	12 (3.8)	
S	Sensory organs	78 (4.2)	17 (5.3)	
V	Various	54 (2.9)	15 (4.7)	
*Originator or generic product*				0.738
Originator		1011 (54.8)	176 (54.3)	
Generic product		833 (45.2)	148 (45.7)	

### Impact—scoring elements

The highest overall score for patient impact was 72 for the shortage of BCG instillation. The second highest ranking was 54 for the shortage of risperidone orodispersible tablets. The shortages of epinephrine injection and methoxy polyethylene glycol-epoetin beta injection both had an impact score of 48. The top five highest-impact shortages included 13 products that had an equal score (Table 2). All shortages with the highest overall patient impact rated moderate impact on alternative products and moderate-to-high impact on the elements “disease” and “costs”.

**Table 2:** Products in shortage with an overall rate on patient impact of 36 or higher

Product	Product characteristics			Rates on the elements of patient impact (rating 1 = low, 2 = moderate, 3 = high)					Overall score
	ATC class	Route of administration	Originator or generic	Alternative product	Disease	Susceptibility	Costs	Number of patients	
BCG instillation	L	Parenteral	Originator	2	3	3	2	2	72
Risperidone orodispersible tablet	N	Oral	Originator	2	3	3	3	1	54
Epinephrine injection	C	Parenteral	Originator	2	3	2	2	2	48
Methoxy polyethylene glycol-epoetin beta injection	B	Parenteral	Originator	2	2	2	3	2	48
Bendamustine powder for infusion	L	Parenteral	Originator	2	3	1	3	2	36
Bromperidol drops for oral use	N	Oral	Originator	2	3	3	2	1	36
Dexamethasone tablet	H	Oral	Generic	2	3	1	3	2	36
Disulfiram dispersible tablet	N	Oral	Originator	2	3	2	3	1	36
Feneticilline capsule	J	Oral	Originator	2	2	1	3	3	36
Flupentixol tablet	N	Oral	Originator	2	3	3	2	1	36
Ibuprofen granules	M	Oral	Originator	2	3	1	2	3	36
Isosorbide mononitrate sustained tablet	C	Oral	Originator	2	3	1	3	2	36
Lamotrigine dispersible tablet	N	Oral	Generic	2	3	2	3	1	36
Olanzapine orodispersible tablet	N	Oral	Generic	2	3	3	2	1	36
Pergolide tablet	N	Oral	Generic	2	3	2	3	1	36
Topiramate tablet	N	Oral	Generic	2	3	2	3	1	36
Vincristine sulfate injection	L	Parenteral	Generic	2	3	1	3	2	36

**Fig. 2:**
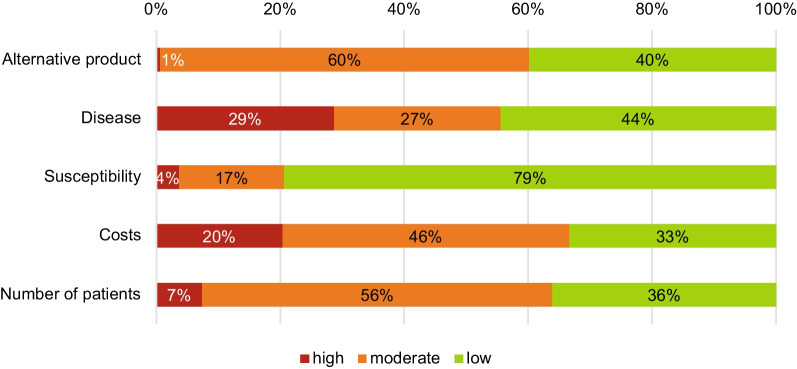
Rates on different elements of patient impact for a sample of medicine shortages in the Netherlands (*n* = 324)

Of the 324 medicine shortages, the elements that frequently received a high impact were disease (29%) and costs (20%) (Fig. 2). Low impact was rated most often for the element susceptibility (79%). The element alternative product had a high impact for only 1% of the shortages and a moderate impact for 60% of the cases.

Nearly half of the evaluated shortages (47%) rated high on one or more elements of patient impact. Nearly 10% of these shortages had high rates on multiple elements. Only 0.3% (*n* = 1) *rated high* on three elements (Additional file 2). None of the shortages rated as high on all five patient impact elements. Only 2% of the shortages rated low impact on all the elements.

### Impact—scoring outcomes of ECHO model

**Fig. 3:**
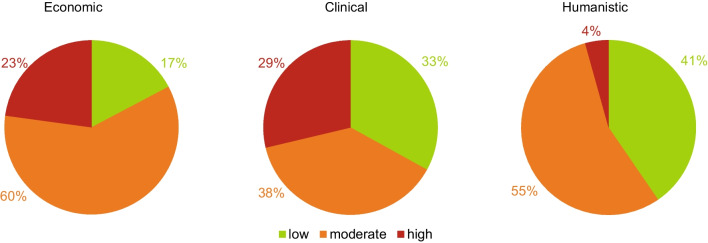
Rates for economic, clinical, and humanistic outcomes (ECHO) for the medicine shortages

Upon converting the rates of the elements to ECHO outcomes, economic outcomes had a high impact in 23% of the cases, clinical outcomes had a high impact in 29%, and humanistic outcomes in 4% (Fig. 3).

### Impact—prevalence and characteristics of products in shortage

**Fig. 4:**
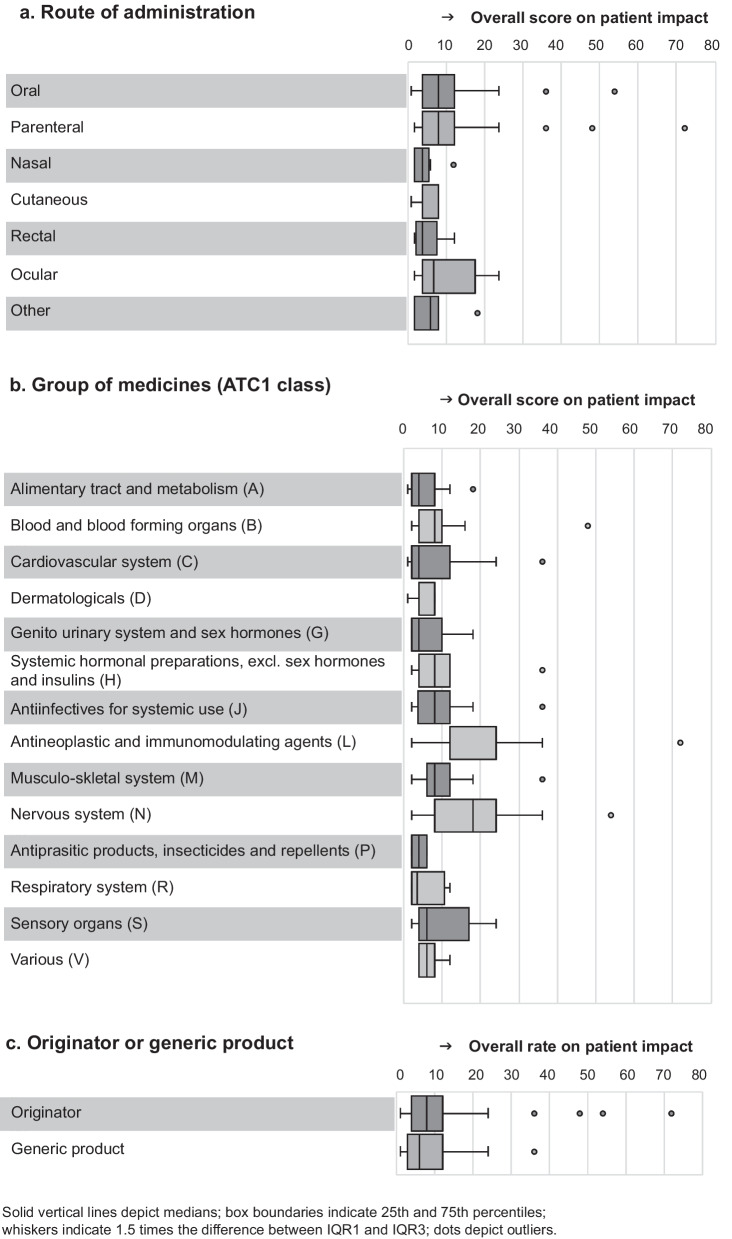
Box and whisker plots depicting relationships between product characteristics and overall scores on patient impact

The median of the overall score for the elements of patient impact was 8 (IQR: 4–12). Oral and parenteral products had similar scores (median score = 8) (Fig. 4a). A significant difference was found between parenteral-nasal/inhalation and parenteral-cutaneous types of medicines. A significant difference between the two most common routes of administration, parenteral and oral, was not found (Additional file 3).

Medicines for the nervous system (ATC class N) had the highest overall patient impact score, with a median of 18 (IQR: 8–24). In addition, the list of products with the highest overall score for patient impact (*n* = 17) consisted of 8 medicines within ATC class N (Table 2). Antineoplastic and immunomodulating agents (ATC class L) also had a higher median overall score for the impact of shortages (median: 12 and IQR: 12–24) than other ATC classes (Fig. 4b). A significant difference between ATC classes N as well as L and 5 out of 13 other therapeutic classes was observed (Additional file 3).

Finally, the overall score for patient impact between originator (median = 8) and generic products (median = 6) showed no statical difference (Fig. 4c).

**Fig. 5:**
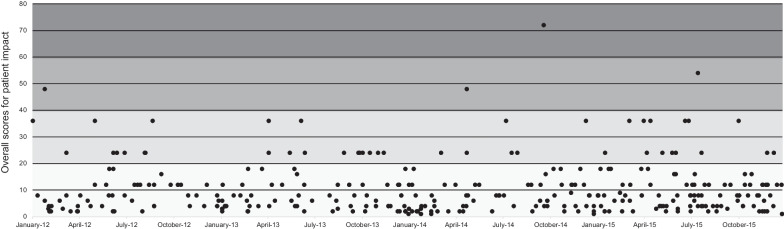
Scores for the overall patient impact of medicine shortages over time

Over time, no pattern in overall scores for patient impact (Fig. 5) was identified. The median of the overall score for patient impact was 8 for 2012, 2014, and 2015 and 6 for the year 2013. However, two elements showed a trend over time. The element “alternative product” showed a decrease in impact: in 2012, this element was rated as having a low impact for 20% of the shortages, and in 2015, 50%. In contrast, the element “costs” showed an increase: in 2012, this element was rated as having a low impact for 48% of the shortages, and in 2015, 27%. The other elements showed slight variation over time (Additional file 4).

## Discussion

This study shows that the impact of medicine shortages on patients is heterogeneous. Various elements were rated as high impact, and the number of elements rated high impact varied substantially between shortages, resulting in different overall impact patterns. Of the five elements that drive patient impact, as described in a previous publication [32], the elements that most frequently rated high were disease (29%) and costs (20%). Nearly half of the evaluated shortages (47%) rated high on at least one element, and nearly 10% of them rated high on multiple elements. When assessing patient outcomes, clinical outcomes were rated as high in 29% of the cases, economic outcomes in 23%, and humanistic outcomes in 4%. High overall impact scores could not significantly be attributed to the characteristics of the products in shortage.

The impact of medicine shortages is often assessed by its direct impact on patients. For instance, at the beginning of a shortage, a (suitable) alternative product is often considered immediately. In the absence of the prescribed treatment for the disease, the impact of shortages on patients is incorporated in models [39–41]. In the current study, this direct impact was rated high in only 30% of the shortages. However, indirect impact—represented by the elements cost, susceptibility, and number of patients—is often overlooked. An additional 17% of the sample’s shortages that rated high on indirect impact were then neglected.

With the increasing number of shortages, stakeholders are trying to identify which shortages necessitate a prompt reaction. For these shortages, a patient cannot miss a dose without negatively impacting their treatment outcome [42]; therefore, all efforts should be made to mitigate the impact. In a previous study [32], it was suggested that these “unforgiving” shortages might have a high rating on multiple elements. Nearly 10% can be considered unforgiving, requiring a prompt reaction from stakeholders.

Product characteristics are often associated with a shortage’s impact. Generally, it is assumed that shortages of parenteral products, antibiotics, and chemotherapies have a high impact on patients since these medicines are difficult to substitute. Although statistics showed that the medians per route of administration were different, the results were inconclusive probably due to small sample size of most groups. However, the sample size of oral and parenteral products was larger, and the assessment of patient impact of these shortages showed no significant difference in overall impact. For the group of medicines, shortages for antineoplastic and immunomodulating agents as well as for medicines for the nervous system showed a higher median score on impact than shortages of medicines for other ATC classes. Their impact was significantly higher for only half of the other therapeutic groups. These medicine groups seem to have a higher impact, but conclusions may be premature.

This is the first study to assess the impact of medicine shortages on clinical, economic, and humanistic outcomes. Until now, the impact of shortages has been based on clinical elements only. Economic and humanistic outcomes have been minimally reported so far, and no study has combined all outcomes [30]. The ECHO-based framework provides an opportunity to combine all these outcomes, creating a more complete overview of the overall impact.

In this study, three limitations may be considered. First, each underlying element of the outcomes of the ECHO model was weighted equally. It can be argued that some elements, such as alternative products, impact patients more than others. However, the advantage of equal weights is that each element is considered. For instance, if a shortage can be solved by a generic substitution, healthcare professionals are likely to regard this solution as having a low patient impact. However, if the generic substitution involves extra costs, the patient impact may become higher.

Second, for calculating the overall impact, the scores for the individual elements were multiplied. Another approach could be to sum up the scores for the five elements. However, the maximum score would then be 15 instead of 243, thus reducing the potential identification of differences. Multiplication is a practical method for highlighting high overall ratings.

Third, the data used concern shortages that started in 2012–2015 in the Netherlands. Since then the Dutch authorities launched the Medicine Shortages and Defects Notification Center in 2017, accompanied by a roadmap describing the various potential solutions for identifying and mitigating the impact of shortages and the roles of various actors (government, manufacturer, wholesale supplier, pharmacist, health insurer) [43]. Together, the notification center and the roadmap aim to quickly identify and resolve shortages. The number of notifications of expected shortages has increased [44] partly due to an increased awareness of the notification obligation among MAHs. However, it is unlikely that the shortage product characteristics are impacted by the rise in notifications of expected shortages. This is confirmed by the consistent pattern observed for the study period (Additional file 5). Moreover, the impact of the shortages, according to the framework, is unlikely to have changed over time because the framework rates the impact of the shortage at the start, irrespective of any measures to mitigate their impact. Furthermore, the center’s ability to prevent supply disruptions is limited.

This study shows a broad view of the impact of medicine shortages. This study also provides insights into the main elements that impact patients, based on shortages in the Netherlands. With increasing numbers of shortages, a tool for the identification of shortages with high-patient impact may be helpful to mitigate their impact on patients in a timely manner. Efforts should target those shortages with the highest patient impact and this framework can be of help to determine the impact of medicine shortages for effective mitigation strategies [31]. We believe that this will be the case regardless of the setting. Individual scores of elements will differ across countries (such as alternative product or costs), but the elements that determine impact will probably be the same or similar. The framework can help stakeholders (healthcare professionals, authorities, and industry) understand the overall impact of medicine shortages on patients and prioritize their efforts to mitigate the impact. Clinical, economic, and humanistic outcomes should be taken into account when determining how the shortage impacts patients, ensuring that the importance of a single outcome type is not being overemphasized at the expense of another type of outcome. Whereas many publications originate from hospital settings, the framework is meant for hospitals and primary care settings. The framework needs application outside our setting for further development to make it robust and applicable in other settings. For example, some of the data sources used for Dutch shortages will need to be replaced, adjusted, or both for another setting.

## Conclusions

Assessing the impact of medicine shortages using a framework based on the ECHO model shows that besides clinical outcomes, economic and humanistic outcomes may have a high impact on patients. This framework can help stakeholders understand the overall impact of medicine shortages on patients. It can also help mitigate the impact on patients by prioritizing the efforts of stakeholders.

## Supplementary Information

**Additional file 1.** Examples of elements influencing the impact of a medicine shortage with increasing impact.

**Additional file 2.** Combination of rates on individual elements for patient impact and their frequency (n (%)).

**Additional file 3.** Comparing overall scores on route of administration as well as ATC classes.

**Additional file 4.** Shortages rated on the elements of patient impact per year (2012-2015).

**Additional file 5.** Characteristics of products in shortage.

## Data Availability

The data on the cohort of the shortages are not publicly available, since data are related to specific products and manufacturers, which is confidential information. Data on costs for medicines as well as age and number of users are third‐party data. These data are not owned by the authors and belong to the Dutch Foundation for Pharmaceutical Statistics (SFK). The SFK welcomes applications from researchers for access to data. Applications are considered by the SFK Supervisory Board. Application forms for data access and further information are available at https://www.sfk.nl/english/foundation‐for‐pharmaceutical‐statistics.
