# Supplementary material for: Medicine shortages: impact behind numbers
Source: J Pharm Policy Pract. 2023 Mar 14;16:44. doi: 10.1186/s40545-023-00548-x (PMC10013985; doi:10.1186/s40545-023-00548-x)
Supplement: Supplementary file 1 — Additional file 1. Examples of elements influencing the impact of a medicine shortage with increasing impact. [file 40545_2023_548_MOESM1_ESM.docx]

# Additional file 1 - Examples of elements influencing the impact of a medicine shortage with increasing impact

| **Rate** | **Alternative product** | | **Disease**  **(disability weight)** | **Susceptibility** | | **Costs** | | **Number of patients affected (% of population)** |
| --- | --- | --- | --- | --- | --- | --- | --- | --- |
|  | *primary aspects* | *secondary aspects* |  | *vulnerability* | *trust in alternative therapy* | *to patients* | *to society* |  |
| Low impact (1 point) | - same substance, licensed,  on-label | - same regimen, strength, concentration or instruction for use - different excipients or labelling | 0 - 0.058 | - 19 - 75 years | full trust | Full reimbursement | no extra medicines costs and/or personnel cost | < 0.05 |
| Moderate impact (2 points) | - different substance, licensed,  on-label - same substance, unlicensed,  on-label - different substance, licensed,  off-label - different substance, unlicensed,  on-label - different substance, unlicensed,  off-label | - interchangeable with extra control - different regimen, strength, concentration or instruction for use or storage | 0.058 -0.224 | - 2 – 18 years, - pregnant and nursing women - patients with divergent metabolism | moderate trust | additional payment | minor to moderate extra medicines costs and/or personnel costs | 0.05 – 0.5 |
| High impact (3 points) | - no or inferior therapy available | - different route of administration | 0.224 – 1 | - < 2 years - > 75 years - patients depending on social care | no trust | full payment | high extra medicines costs and/or personnel costs | > 0.5 |
