# Supplementary material for: Medicine shortages: impact behind numbers
Source: J Pharm Policy Pract. 2023 Mar 14;16:44. doi: 10.1186/s40545-023-00548-x (PMC10013985; doi:10.1186/s40545-023-00548-x)
Supplement: Supplementary file 2 — Additional file 2. Combination of rates on individual elements for patient impact and their frequency (n (%)). [file 40545_2023_548_MOESM2_ESM.docx]

**Additional file 2 - Combination of rates on individual elements for patient impact and their frequency (n (%))**

n 1 1 13 15 2 2 28 55 25 10 8 51 59 47 7

% (0,3) (0,3) (4,0) (4,6) (0,6) (0,6) (8,6) (17,0) (7,7) (3,1) (2,5) (15,7) (18,2) (14,5) (2,2)
