# Supplementary material for: Medicine shortages: impact behind numbers
Source: J Pharm Policy Pract. 2023 Mar 14;16:44. doi: 10.1186/s40545-023-00548-x (PMC10013985; doi:10.1186/s40545-023-00548-x)
Supplement: Supplementary file 3 — Additional file 3. Comparing overall scores on route of administration as well as ATC classes. [file 40545_2023_548_MOESM3_ESM.docx]

# Additional file 3 - Comparing overall scores on route of administration as well as ATC classes

**Table: Mann-Whitney U test results for Kruskal-Wallis analysis of variance on route of administration**(grey background: significant differences (p < 0.05) between two routes of administration)

|  | Oral | Parenteral | Nasal/ inhalation | Cutaneous | Rectal | Ocular | Other |
| --- | --- | --- | --- | --- | --- | --- | --- |
| Oral | - | .420 | .051 | .079 | .292 | .937 | .254 |
| Parenteral |  | - | .008 | .008 | .096 | .603 | .117 |
| Nasal/inhalation |  |  | - | .414 | .481 | .076 | .613 |
| Cutaneous |  |  |  | - | .948 | .170 | .938 |
| Rectal |  |  |  |  | - | .379 | .837 |
| Ocular |  |  |  |  |  | - | .368 |
| Other |  |  |  |  |  |  | - |

**Table: Dunn test results for Kruskal-Wallis analysis of variance on ATC class**(grey background: significant differences (p < 0.05) between two ATC-classes)

|  | Alimentary tract and metabolism (A) | Blood and blood forming organs (B) | Cardiovascular system (C) | Dermatologicals (D) | Genito urinary system and sex hormones (G) | Systemic hormonal preparations, excl. sex hormones and insulins (H) | Antiinfectives for systemic use (J) | Antineoplastic and immunomodulating  agents (L) | Musculo-skletal system (M) | Nervous system (N) | Antiprasitic products, insecticides and  repellents (P) | Respiratory system (R) | Sensory organs (S) | Various (V) |
| --- | --- | --- | --- | --- | --- | --- | --- | --- | --- | --- | --- | --- | --- | --- |
| Alimentary tract and metabolism (A) | - | 1.000 | 1.000 | 1.000 | 1.000 | 1.000 | 1.000 | .000 | 1.000 | .000 | 1.000 | 1.000 | 1.000 | 1.000 |
| Blood and blood forming organs (B) |  | - | 1.000 | 1.000 | 1.000 | 1.000 | 1.000 | 1.000 | 1.000 | .549 | 1.000 | 1.000 | 1.000 | 1.000 |
| Cardiovascular system (C) |  |  | - | 1.000 | 1.000 | 1.000 | 1.000 | .000 | 1.000 | .000 | 1.000 | 1.000 | 1.000 | 1.000 |
| Dermatologicals (D) |  |  |  | - | 1.000 | 1.000 | 1.000 | .008 | 1.000 | .001 | 1.000 | 1.000 | 1.000 | 1.000 |
| Genito urinary system and sex hormones (G) |  |  |  |  | - | 1.000 | 1.000 | .052 | 1.000 | .007 | 1.000 | 1.000 | 1.000 | 1.000 |
| Systemic hormonal preparations, excl. sex hormones and insulins (H) |  |  |  |  |  | - | 1.000 | 1.000 | 1.000 | 1.000 | 1.000 | 1.000 | 1.000 | 1.000 |
| Antiinfectives for systemic use (J) |  |  |  |  |  |  | - | .003 | 1.000 | .000 | 1.000 | 1.000 | 1.000 | 1.000 |
| Antineoplastic and immunomodulating agents (L) |  |  |  |  |  |  |  | - | 1.000 | 1.000 | .875 | .004 | 1.000 | .271 |
| Musculo-skletal system (M) |  |  |  |  |  |  |  |  | - | 1.000 | 1.000 | 1.000 | 1.000 | 1.000 |
| Nervous system (N) |  |  |  |  |  |  |  |  |  | - | .564 | .000 | .673 | .053 |
| Antiprasitic products, insecticides and repellents (P) |  |  |  |  |  |  |  |  |  |  | - | 1.000 | 1.000 | 1.000 |
| Respiratory system (R) |  |  |  |  |  |  |  |  |  |  |  | - | 1.000 | 1.000 |
| Sensory organs (S) |  |  |  |  |  |  |  |  |  |  |  |  | - | 1.000 |
| Various (V) |  |  |  |  |  |  |  |  |  |  |  |  |  | - |
