# Supplementary figures and images for: Medicine shortages: impact behind numbers
Source: J Pharm Policy Pract. 2023 Mar 14;16:44. doi: 10.1186/s40545-023-00548-x (PMC10013985; doi:10.1186/s40545-023-00548-x)

**Additional file - Shortages rated on the elements of patient impact per year (2012-2015)**


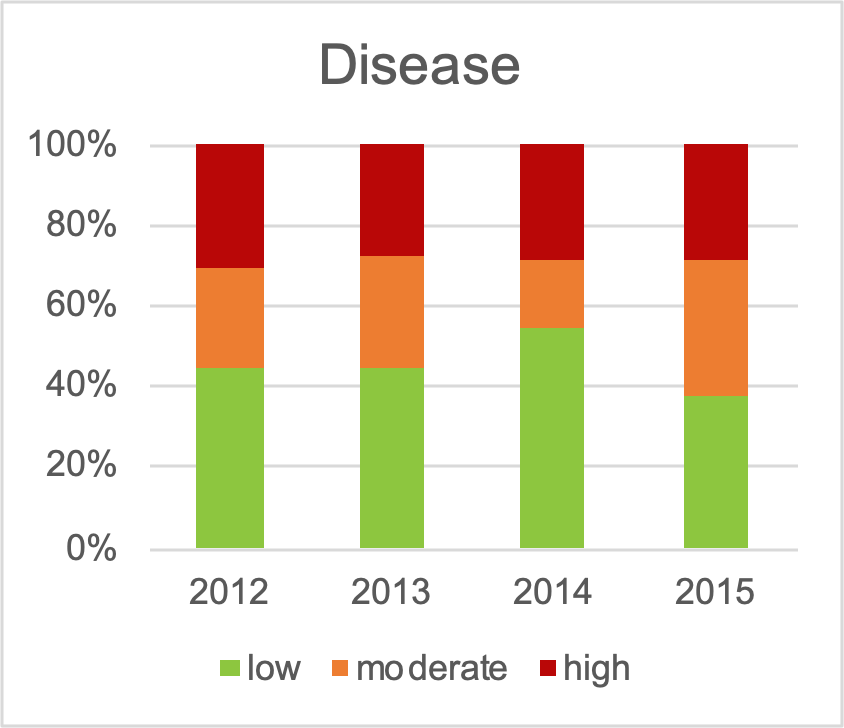

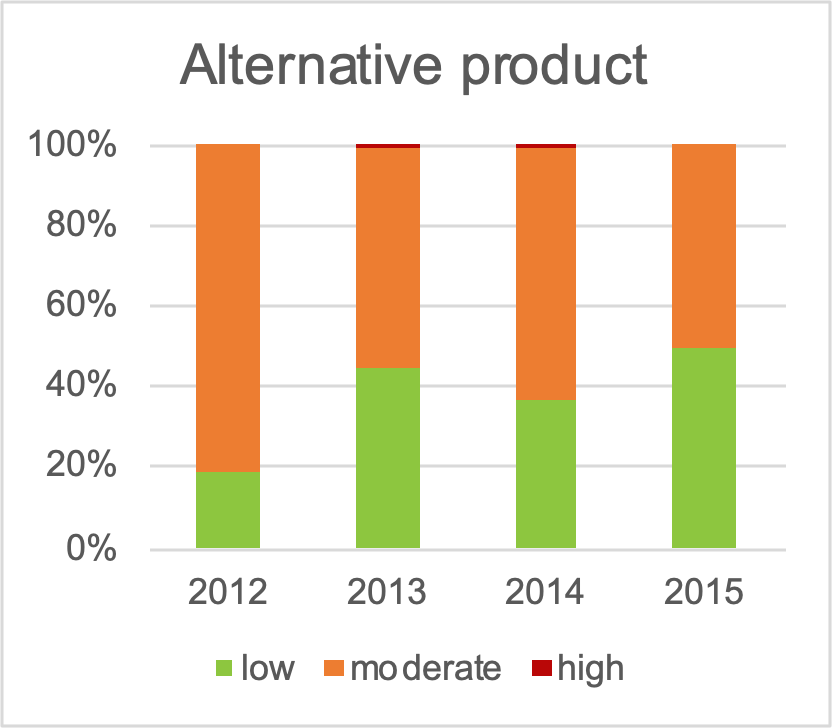


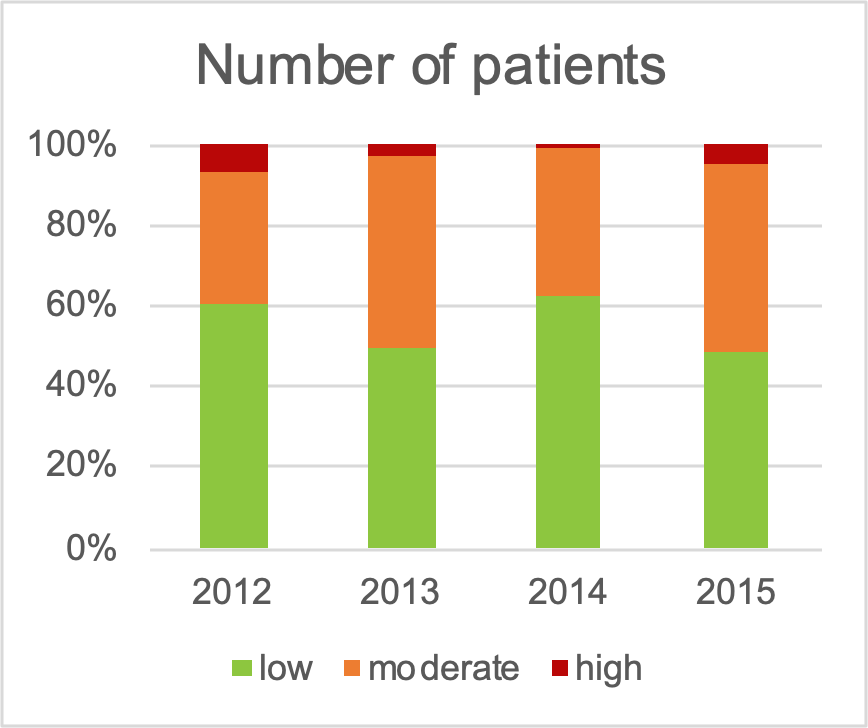

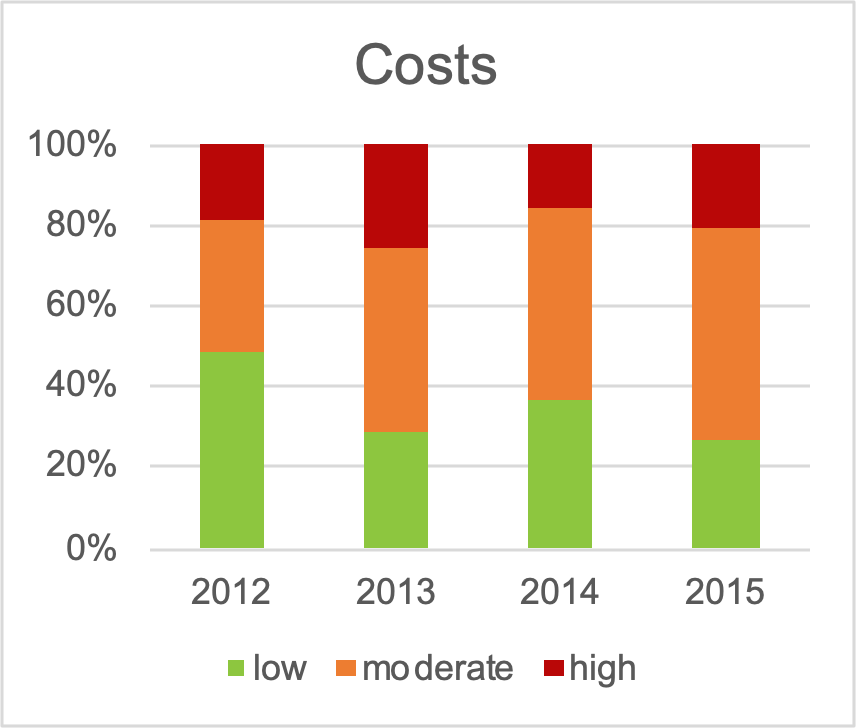

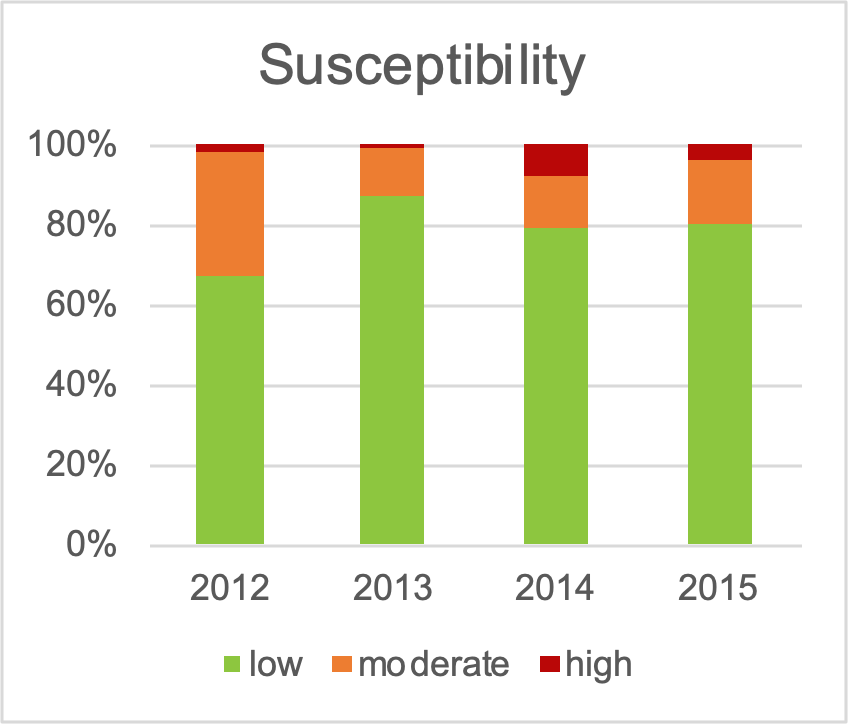

Supplement: Supplementary file 4 — Additional file 4. Shortages rated on the elements of patient impact per year (2012-2015). [file 40545_2023_548_MOESM4_ESM.docx]
