# Supplementary material for: Medicine shortages: impact behind numbers
Source: J Pharm Policy Pract. 2023 Mar 14;16:44. doi: 10.1186/s40545-023-00548-x (PMC10013985; doi:10.1186/s40545-023-00548-x)
Supplement: Supplementary file 5 — Additional file 5. Characteristics of products in shortage. [file 40545_2023_548_MOESM5_ESM.docx]

# Supplement S5 - Characteristics of products in shortage

|  | | | **2012** | **2013** | **2014** | **2015** |
| --- | --- | --- | --- | --- | --- | --- |
| n = 1,844 | | | n = 387 | n = 439 | n = 470 | n = 548 |
| **Route of administration** | | |  |  |  |  |
|  | Oral | | 212 | 232 | 276 | 325 |
|  | Parenteral | | 138 | 145 | 122 | 149 |
|  | Nasal/inhalation | | 2 | 16 | 10 | 14 |
|  | Cutaneous | | 13 | 12 | 24 | 28 |
|  | Rectal | | 2 | 8 | 12 | 3 |
|  | Ocular | | 18 | 12 | 16 | 19 |
|  | Other | | 2 | 14 | 10 | 9 |
| **Group of medicines (ATC1 class)** | | |  |  |  |  |
|  | A | Alimentary tract and metabolism | 38 | 36 | 50 | 44 |
|  | B | Blood and blood forming organs | 27 | 23 | 18 | 23 |
|  | C | Cardiovascular system | 46 | 48 | 82 | 64 |
|  | D | Dermatologicals | 11 | 11 | 27 | 34 |
|  | G | Genito urinary system and sex hormones | 18 | 28 | 13 | 18 |
|  | H | Systemic hormonal preparations, excl. sex hormones and insulins | 9 | 25 | 27 | 22 |
|  | J | Antiinfectives for systemic use | 62 | 89 | 53 | 107 |
|  | L | Antineoplastic and immunomodulating agents | 39 | 38 | 37 | 33 |
|  | M | Musculo-skletal system | 21 | 16 | 15 | 33 |
|  | N | Nervous system | 76 | 73 | 88 | 96 |
|  | P | Antiprasitic products, insecticides and repellents |  |  | 2 | 2 |
|  | R | Respiratory system | 6 | 23 | 27 | 32 |
|  | S | Sensory organs | 19 | 19 | 18 | 22 |
|  | V | Various | 14 | 10 | 13 | 17 |
| **Originator or generic product** | | |  |  |  |  |
|  | Originator | | 182 | 191 | 200 | 260 |
|  | Generic product | | 205 | 248 | 270 | 287 |
